# Water Hardness Improves the Antioxidant Response of Zinc-Exposed Goldfish (*Carassius auratus*)

**DOI:** 10.3390/biology12020289

**Published:** 2023-02-10

**Authors:** Cheol Young Choi, Min Ju Kim, Jin Ah Song, Kang Hee Kho

**Affiliations:** 1 Division of Marine BioScience, Korea Maritime and Ocean University, Busan 49112, Republic of Korea; 2 Department of Convergence Study on the Ocean Science and Technology, Korea Maritime and Ocean University, Busan 49112, Republic of Korea; 3 Marine Bio-Resources Research Unit, Korea Institute of Ocean Science and Technology, Busan 49111, Republic of Korea; 4 Department of Fisheries Science, Chonnam National University, Yeosu 59626, Republic of Korea

**Keywords:** antioxidant response, goldfish, oxidative stress, water hardness, zinc

## Abstract

**Simple Summary:**

Zinc (Zn), known as a heavy metal and an essential element, is beneficial to fish in small amounts, but exposure to high concentrations of Zn induces physiological changes in fish. These changes may disrupt homeostasis in fish and cause stress. The water hardness of fresh water has the effect of reducing stress caused by Zn. In this study, to investigate the effect of water hardness on zinc-induced oxidative stress, we confirmed the extent of oxidative stress and apoptosis by exposing goldfish to various Zn concentrations and water hardness. Ultimately, our results suggest that a high water hardness level decreases Zn-induced toxicity, which is expected to be able to provide new insight into our understanding of how aquatic environmental factors, such as water hardness, affect the maintaining of the antioxidant system of fish.

**Abstract:**

Zinc (Zn), a heavy metal, is an essential element in fish; however, exposure to high concentrations causes oxidative stress. Water hardness reduces oxidative stress reactions caused by heavy metals. To confirm the effect of water hardness on oxidative stress caused by Zn, goldfish were exposed to various Zn concentrations (1.0, 2.0, and 5.0 mg/L) and water hardness (soft (S), hard (H), and very hard (V)). The activity of superoxide dismutase (SOD) and catalase (CAT) in plasma increased with 1.0, 2.0, and 5.0 mg/L of Zn, and decreased with H and V water hardness. The levels of H_2_O_2_ and lipid peroxide (LPO) increased with Zn above 1.0 mg/L and decreased with H and V of water hardness. Caspase-9 mRNA expression in the liver increased after 7 and 14 days of Zn exposure and decreased with H and V water hardness. It was confirmed that DNA damage was less dependent on H and V water hardness. Based on the results of this study, at least 1.0 mg/L Zn causes oxidative stress in goldfish, and a high level of apoptosis occurs when exposed for more than 7 days. It appears that the oxidative stress generated by Zn can be alleviated by water hardness of at least 270 mg/L CaCO_3_. This study provides information on the relationship between the antioxidant response caused by heavy metals and water hardness in fish.

## 1. Introduction

The amount of various pollutants discharged into the ecosystem is increasing as a result of ongoing industrial activities, and discharged pollutants have adverse effects on aquatic ecosystems [1]. Among these pollutants, the concentration of heavy metals in aquatic ecosystems is increasing owing to industrialization and urbanization, which is a major issue [2]. Heavy metals are transferred to higher predators via the food chain because they are not biologically degradable and are not easily excreted after accumulating in the bodies of aquatic organisms [3]. In addition, heavy metals have been reported to be toxic to fish and cause tissue damage and oxidative stress [4]. High levels of heavy metals are present in almost all aquatic ecosystems worldwide [5]. Among the various heavy metals, zinc (Zn) is known to exist at high levels in freshwater ecosystems. In fact, it has been confirmed that the Ganges River in India contains up to 0.122 ppm [6], and the Han River in Korea contains up to 0.6 ppm of it [7].

Although Zn is an essential trace element for fish, excess Zn is known to have adverse effects on fish [8]. When an organism is exposed to high concentrations of heavy metals such as Zn, it undergoes oxidative stress, and reactive oxygen species (ROS) such as superoxide radical anions (O_2_^−^), hydrogen peroxide (H_2_O_2_), and hydroxyl radicals (OH^−^) are excessively produced [9]. Although ROS are generated by metabolism and cellular activity, excessive ROS cause tissue damage by oxidizing lipids or DNA [10].

Therefore, to stabilize the generation of ROS induced by oxidative stress, organisms induce antioxidant reactions, such as increasing the activity of antioxidant genes in the body, thereby balancing the generation of ROS and antioxidant reactions to maintain homeostasis in the body [11]. Antioxidant enzymes are expressed in all tissues where an antioxidant reaction occurs; however, in fish, they are mainly expressed in the liver tissues [12].

Superoxide dismutase (SOD) and catalase (CAT) are well-known antioxidant enzymes, as well as glutathione peroxidase (GPX) and glutathione reductase (GSR) [13]. According to the structure of the enzyme active site and metal cofactors, SOD is classified into manganese SOD (Mn-SOD) and copper/zinc SOD (Cu/Zn-SOD) [14]. It is well known that Mn-SOD can effectively remove O_2_^−^ produced as a by-product of oxidative phosphorylation [15].

As a representative antioxidant reaction caused by antioxidant enzymes, SOD converts O_2_^−^, an active oxygen produced in tissues, into H_2_O_2_, and CAT converts H_2_O_2_ into non-toxic H_2_O and O_2_ [16]. However, residual ROS due to an insufficient capacity of the antioxidant process to ensure a balance between production and removal of ROS, oxidize membrane lipids and induce a lipid peroxidation reaction that raises the level of lipid peroxide (LPO), which is used as a representative biomarker for detecting oxidative stress [17]. In addition, DNA damage occurs because of cell membrane damage caused by lipid peroxidation as well as DNA and protein denaturation [18]. In addition, ROS oxidize signaling proteins, thereby activating the progression of apoptosis through caspase-9 [19].

Although Zn is an essential trace element for fish, it is often toxic when it enters freshwater ecosystems in large quantities, mainly through wastewater and soil pollution, along with other hazardous products. It is known that water hardness (Ca^2+^ ions in water) plays a role in protecting fish from Zn toxicity [20,21]. Therefore, in this study, goldfish, which are mainly used for toxicity evaluation owing to their low sensitivity to toxic substances, were exposed to various concentrations of Zn and water hardness. The changes in oxidative stress and antioxidant responses of goldfish in response to Zn exposure and water hardness were investigated. The activity of antioxidant enzymes, concentration of active oxygen, and degree of lipid peroxidation were confirmed. In addition, to confirm the effect of the combination of Zn and water hardness on apoptosis in goldfish liver, caspase-9 mRNA expression analysis and comet assay were performed.

## 2. Materials and Methods

### 2.1. Experiment Fishes

Goldfish *Carassius auratus* (body length 6.8 ± 0.8 cm; mass 12.4 ± 2.1 g), an experimental organism, were purchased from Choryang Aquarium (Busan, Republic of Korea) and acclimated for 7 days in six 300 L fresh water tanks in the laboratory. The fresh water was kept at 19.5 ± 1.1 °C and pH 7.8 with constant aeration. Feeding was stopped 24 h prior to the experiment.

### 2.2. Zinc and Water Hardness Treatment and Sampling

The Zn used in the experiment was ZnSO_4_ (83265, Sigma, St. Louis, MO, USA), and the goldfish used in the experiment were exposed to different Zn concentrations (0, 1.0, 2.0, and 5.0 mg/L) and water hardness (soft, hard, and very hard). The Zn concentrations were set by referring to a previous study [6,7,22].

Water hardness was set by adding CaCO_3_ (239216, Sigma, St. Louis, MO, USA). The water hardness of the control group was 90 mg/L CaCO_3_, which was established with reference to the water hardness value of running water in Busan, Korea. Water hardness was measured using a pure water meter (PWH-303, Lutron, wanchai, Hong Kong, China). We created three water hardness conditions: soft water (S, CaCO_3_: 90 mg/L), hard water (H, CaCO_3_: 270 mg/L), and very hard water (V, CaCO_3_: 450 mg/L).

We named the experiment groups as shown below for convenience: the control group was set as Zn 0 + S (0 mg/L Zn in soft water), other experiment groups were set as Zn 1.0 + S (1.0 mg/L Zn in soft water), Zn 2.0 + S (2.0 mg/L Zn in soft water), Zn 5.0 + S (5.0 mg/L Zn in soft water), Zn 1.0 + H (1.0 mg/L Zn in hard water), Zn 2.0 + H (2.0 mg/L Zn in hard water), Zn 5.0 + H (5.0 mg/L Zn in hard water), Zn 1.0 + V (1.0 mg/L Zn in very hard water), Zn 2.0 + V (2.0 mg/L Zn in very hard water), and Zn 5.0 + V (5.0 mg /L Zn in very hard water).

During this experiment, water hardness was measured daily and recorded. All fish were anesthetized with clove oil (C8392, Sigma, St. Louis, MO, USA) before sampling. Blood was rapidly collected from the caudal vein using an injector coated with heparin. Plasma was collected by separating blood using centrifugation (4 °C at 12,000× *g* for 12 min). Liver tissue samples were fixed at room temperature in 4% paraformaldehyde (PFA) prior to analysis of in situ hybridization.

### 2.3. Total RNA Extraction and Complementary DNA Synthesis

Total RNA from the liver tissue was extracted using TRI Reagent^®^ (TR188, Molecular Research Center, Cincinnati, OH, USA) according to the manufacturer’s instructions. The purity (A260/A280) of all RNA was calculated to be approximately 1.8–2.0. The total RNA (2 μg) was reverse-transcribed to complementary DNA (cDNA). Reverse transcription was performed using an oligo-(dT)_15_ anchor and M-MLV reverse transcriptase (RT0015, Takara, Japan) according to the manufacturer’s instructions. All the synthesized cDNA was stored at −20 °C and diluted by 1:100 before the real-time polymerase chain reaction.

### 2.4. Real-Time Polymerase Chain Reaction

The relative expression of caspase-9 and β-actin mRNA was measured using real-time quantitative polymerase chain reaction (qPCR). The qPCR primers used in the experiment were designed according to sequences in the NCBI database (Table 1). A Bio-Rad iCycler iQ multicolor real-time PCR detection system (Bio-Rad, Hercules, CA, USA) and iQ SYBR green supermix (Bio-Rad, Hercules, CA, USA) were used for qPCR amplification in accordance with the manufacturer’s instructions. The β-actin used in the experiment is an internal control for comparing the relative mRNA expression level. All data were expressed as changes with respect to the corresponding β-actin-calculated cycle threshold (ΔCt) levels. The calibrated ΔCt value (ΔΔCt) for each sample and internal control (β-actin) was calculated using the 2^−ΔΔCt^ method.

### 2.5. In Situ Hybridization Detection of SOD mRNA

The SOD sequence for the in situ hybridization probe was designed as shown in Table 1. Then, using PCR amplification, it was amplified and ligated into the pGEM-T easy vector (A137A, Promega, Madison, WI, USA). The antisense was confirmed using PCR amplification of the plasmid DNA with the antisense, sense, and T7 primers (5′-TAA TAC GAC TCA CTA TAG GG-3′). Digoxigenin (DIG)-labelled RNA probes were created using a DIG RNA Labeling Mix (Merck, Darmstadt, Germany), and the PCR products using the anti-sense primer and T7 RNA polymerase (Merck, Darmstadt, Germany) were used as the anti-sense labelling probes.

After 14 days of exposure, the liver tissues of fish (Zn 0 + S (control), Zn 5.0 + S, Zn 5.0 + H, and Zn 5.0 + V) were fixed in 4% PFA overnight at 4 °C. After fixation, the samples were washed with PBS and stored in 30% sucrose to prevent frostbite. The sections were hybridized with hybridization buffer (25 mL deionized formamide, 12.5 mL of 20× saline sodium citrate, 500 μL 0.1% Tween-20, 460 μL 1 M citric acid (pH 6.0), and DEPC-H_2_O up to a total volume of 50 mL), yeast total RNA (50 μL per 950 μL hybridization buffer), and the RNA probe overnight at 65 °C.

For hybridization signal detection, the tissue sections were first incubated with a blocking solution (containing 10% calf serum in PBST) for 1 h at room temperature. The tissue sections were then incubated at 4 °C with an alkaline phosphatase-conjugated anti-digoxigenin antibody (1:2000 in blocking solution; Roche, Basel, Switzerland) after a series of washing steps (six times for 15 min each, in PBST at room temperature) and rinsing in alkaline Tris buffer (1 M Tris at pH 9.5, 1 M MgCl_2_, 5 M NaCl, 10% Tween-20) three times for 5 min each at room temperature. Color development was performed by spraying over the sections using a labelling mix (1 mL alkaline Tris buffer, 4.5 μL nitroblue tetrazolium, and 3.5 μL 5-bromo-4-chloro-3-indolyl phosphate disodium salt). The tissue was then stored in a dark and humid chamber for at least 1 h for color development. When color was detected, slides were washed with PBST approximately three times, mounted with Aquamount (Aqua Polymount, Warrington, PA, USA), and covered with a slide cover glass. A stereomicroscope (Nikon Eclipse Ci, Tokyo, Japan) was used to capture the images.

### 2.6. Analysis of Plasma Parameter

Following the manufacturer’s instructions, SOD and CAT activities were measured using an ELISA kit (SOD; MBS705758, CAT; MBS705697, Mybiosource, San Diego, CA, USA), and the absorbance was measured at 450 nm. An ELISA kit (MBS1601700, Mybiosource, San Diego, CA, USA) was used to measure LPO levels in accordance with the manufacturer’s instructions, and absorbance was measured at 540 nm. The H_2_O_2_ levels were measured using a peroxidase activity assay kit (MAK092, Sigma-Aldrich, Burlington, NJ, USA) according to the manufacturer’s instructions. The absorbance of the H_2_O_2_ sample was measured at 540 nm wavelength.

### 2.7. Comet Assays

The comet assay is a sensitive technique for quantitative measurement of DNA damage in cells. Digestive diverticula cells (1 × 10^5^ cells/mL) were examined using a Comet Assay Reagent kit with single-cell gel electrophoresis assays (Trevigen Inc., Minneapolis, MD, USA) according to the method described by Singh et al. [23], with some modifications. Cells were immobilized on agarose gels on comet slides and immersed in an alkaline unwinding solution for 20 min in the dark. The slides were then electrophoresed at 21 V for 30 min with an alkaline electrophoresis solution and dried for 15 min at room temperature. Samples were stained with SYBR Gold solution (Trevigen Inc., Minneapolis, MN, USA) for 30 min in the dark. Images were captured using a fluorescence microscope (excitation filter 465–495 nm; Nikon Eclipse Ci, Tokyo, Japan). We confirmed that at least 100 cells were analyzed from each slide. To quantify the comet assay results, we analyzed the tail length (distance of DNA migration from the head) and percentage of DNA in the tail (tail intensity/total intensity) using the comet assay IV image analysis software (version 4.3.2; Perceptive Instruments Ltd., Suffolk, England, UK).

### 2.8. Statistical Analysis

All data were analyzed using SPSS version 25.0 (IMB SPSS Inc., Armonk, NY, USA). For all the parameters analyzed, two-way ANOVA followed by Tukey’s post hoc test was used to compare differences between the different Zn levels, water hardness, and different times of the sample in the data. The significance level was greater than 95% (*p* < 0.05). Values were expressed as the mean ± standard deviation (SD).

## 3. Results

### 3.1. Water Hardness

The water hardness of each experimental group during experiment were shown in Table 2. Water hardness values indicate means ± standard deviation (*n* = 5). In the soft water group with different Zn concentrations, a water hardness of approximately 90 mg/L CaCO_3_ was maintained. Similarly, the hard water and very hard water groups retained approximately 270 and 450 mg/L CaCO_3_, respectively.

### 3.2. Changes SOD and CAT Activity in Plasma

**Figure 1:**
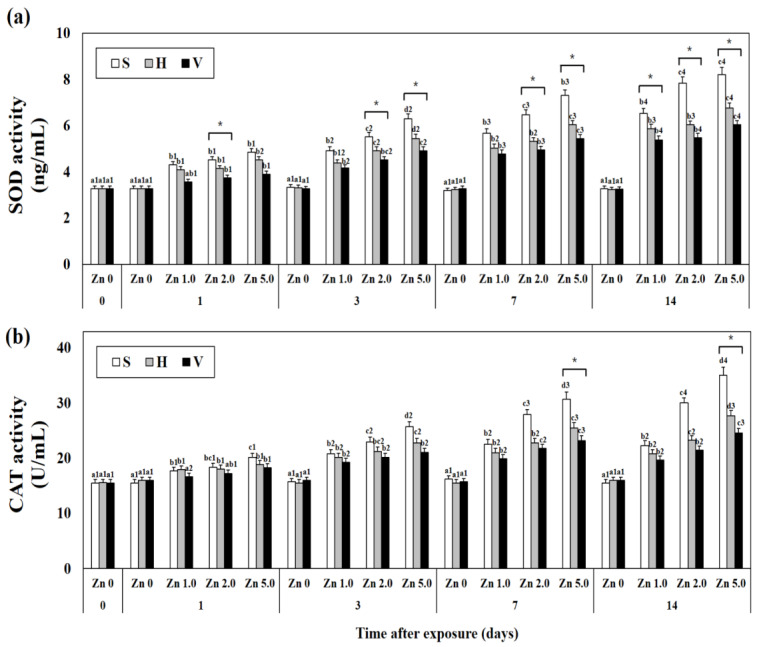
Changes in the (**a**) SOD and (**b**) CAT activity measured for 14 days. Different letters indicate significant differences among goldfish exposed to the same water hardness and different Zn concentration for same duration (*p* < 0.05). Different numbers indicate significant differences among goldfish exposed to same Zn concentrations and water hardness for the different exposure time (*p* < 0.05). “*” indicates significant differences among goldfish exposed to different water hardness and same Zn concentration for same duration (*p* < 0.05). “*” was marked when there were only significant differences in all combinations of the values of each S/H/V experimental group. Values indicate means ± SD (*n* = 4).

Changes in plasma SOD and CAT activities with different Zn concentrations and water hardness were investigated (Figure 1). As Zn concentration and the number of exposure days increased, SOD activity also increased. When comparing different water hardness values in each experimental group set with the same exposure day and Zn concentration, the activity was generally lower as water hardness increased (i.e., day 1: Zn 5.0 + S; 4.85 ± 0.16, Zn 5.0 + H; 4.51 ± 0.14, Zn 5.0 + V; 3.90 ± 0.13, *p* < 0.05). In addition, compared with the control group, no change in SOD and CAT activities due to different water hardness (S: 90 mg/L CaCO_3_, H: 240 mg/L CaCO_3_, V: 450 mg/L CaCO_3_) was observed in Zn 0 concentration groups.

Similar to SOD activity, CAT activity was found to increase as Zn concentration and the number of exposure days increased. When comparing different water hardness values in each experimental group set with the same exposure day and Zn concentration, the activity was generally lower as water hardness increased (i.e., day 1: Zn 5.0 + S; 20.16 ± 0.72, Zn 5.0 + H; 18.86 ± 0.68, Zn 5.0 + V; 18.30 ± 0.67, *p* < 0.05). Similarly, compared with the control group, no change in mRNA expression with different water hardness was observed in Zn 0 concentration groups.

### 3.3. SOD mRNA Expression in Liver Using In Situ Hybridization

**Figure 2:**
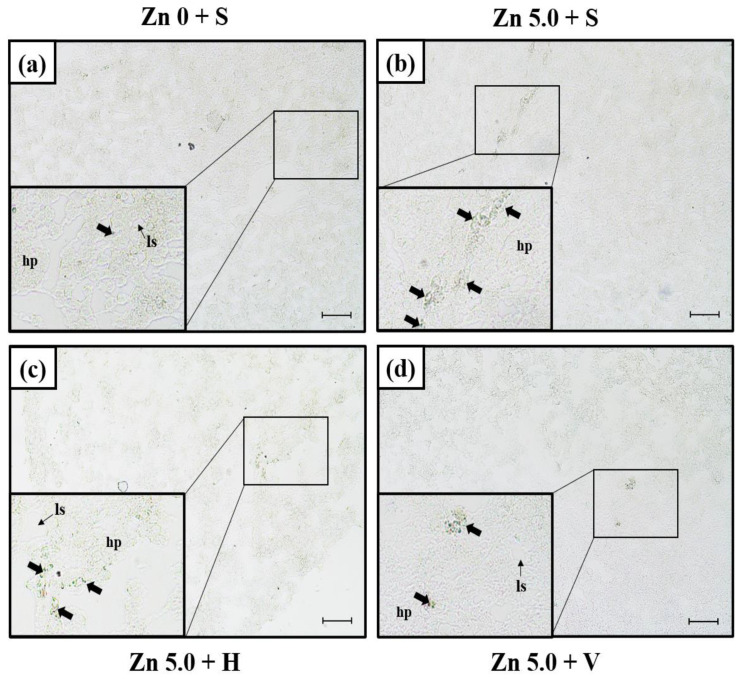
Detection of SOD mRNA expression in liver of goldfish by in situ hybridization: (**a**) Zn 0 + S (control) expression site and magnification of mRNA expression site in (**a**); (**b**) Zn 5.0 + S expression site and magnification of mRNA expression site in (**b**); (**c**) Zn 5.0 + H expression site and magnification of mRNA expression site in (**c**); (**d**) Zn 5.0 + V expression site and magnification of mRNA expression site in (**d**). The dark area (black arrow) indicates the expression of SOD mRNA in liver (scale bars = 50 μm). Abbreviation: hp; hepatocytes, ls; liver sinusoid.

The expression of SOD mRNA in the liver was analyzed using in situ hybridization (Figure 2). The presence of SOD mRNA expression was confirmed near the nucleus of hepatocytes in liver tissue. For 14 days, the SOD mRNA expression signals in the control group were fewer than those in the Zn exposure groups (Zn 5.0 + S, Zn 5.0 + H, Zn 5.0 + V). The Zn 5.0 + S group showed more signals than the Zn 5.0 + H and Zn 5.0 +V groups. The intensive signal of SOD mRNA was observed in Zn 5.0 + V compared with that in Zn 5.0 + H. The scale bar size was 50 μm.

### 3.4. Levels of Hydrogen Peroxide and Lipid Peroxidation in Plasma

**Figure 3:**
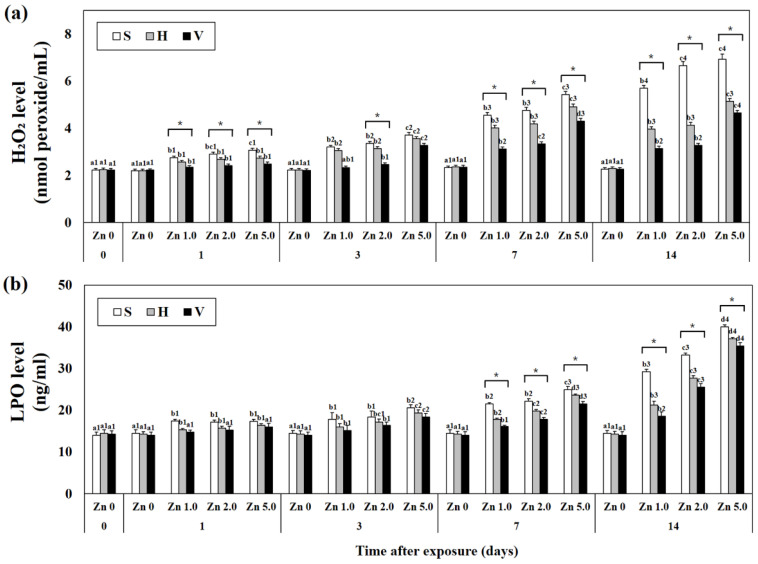
Concentration of (**a**) H_2_O_2_ and (**b**) LPO in plasma of goldfish for 14 days. Different letters indicate significant differences among goldfish exposed to the same water hardness and different Zn concentration for same duration (*p* < 0.05). Different numbers indicate significant differences among goldfish exposed to same Zn concentrations and water hardness for the different exposure time (*p* < 0.05). “*” indicates significant differences among goldfish exposed to different water hardness and same Zn concentration for same duration (*p* < 0.05). “*” was marked when there were only significant differences in all combinations of the values of each S/H/V experimental group. Values indicate means ± SD (*n* = 4).

Plasma H_2_O_2_ levels tended to increase with increasing Zn concentrations and days of Zn exposure (Figure 3). When H_2_O_2_ levels were compared based on the same concentration of Zn in each exposure group, the concentration decreased with increasing water hardness (i.e., day 14: Zn 5.0 + S; 6.93 ± 0.21, Zn 5.0 + H; 5.14 ± 0.10, Zn 5.0 + V; 4.65 ± 0.09, *p* < 0.05). In addition, compared to the control group, no change in H_2_O_2_ levels due to water hardness was observed in Zn 0 concentration groups (i.e., day 14: Zn 0 + S; 2.26 ± 0.6, Zn 0 + H; 2.30 ± 0.07, Zn 0 + V; 2.26 ± 0.06, *p* < 0.05).

Plasma LPO levels increased with increasing Zn concentrations and days of Zn exposure. When comparing the LPO level based on the same Zn concentration, low activity was found with high water hardness (i.e., day 14: Zn 1.0 + S; 29.25 ± 0.58, Zn 1.0 + H; 21.26 ± 0.92, Zn 1.0 + V; 18.54 ± 1.07, *p* < 0.05). Similar to the change in H_2_O_2_ levels, no significant change in LPO levels due to water hardness was observed in Zn 0 concentration groups.

### 3.5. Changes of Caspase-9 mRNA Expression in Liver

**Figure 4:**
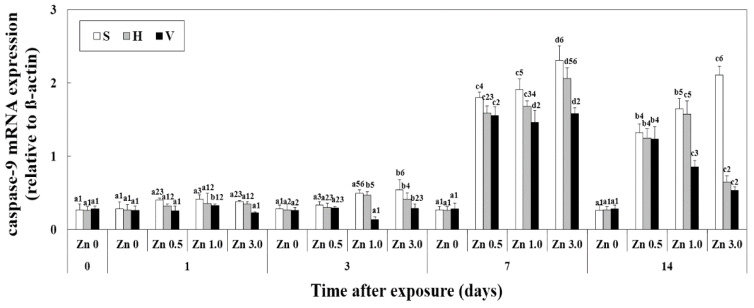
Changes in the mRNA expression of caspase-9 measured for 14 days. Different letters indicate significant differences among goldfish exposed to the same water hardness and different Zn concentration for same duration (*p* < 0.05). Different numbers indicate significant differences among goldfish exposed to same Zn concentrations and water hardness for the different exposure time (*p* < 0.05).

The expression of caspase-9 mRNA in the liver at different Zn concentrations and water hardness levels was analyzed using qPCR (Figure 4).

When compared to day 1 and day 3, the expression of caspase-9 mRNA involved in apoptosis was significantly increased on days 7 and 14. The expression also decreased as water hardness increased (i.e., day 14: Zn 5.0 + S; 1.21 ± 0.04, Zn 5.0 + H; 0.92 ± 0.06, Zn 5.0 + V; 0.75 ± 0.07, *p* < 0.05).

### 3.6. Analysis of DNA Damage

**Figure 5:**
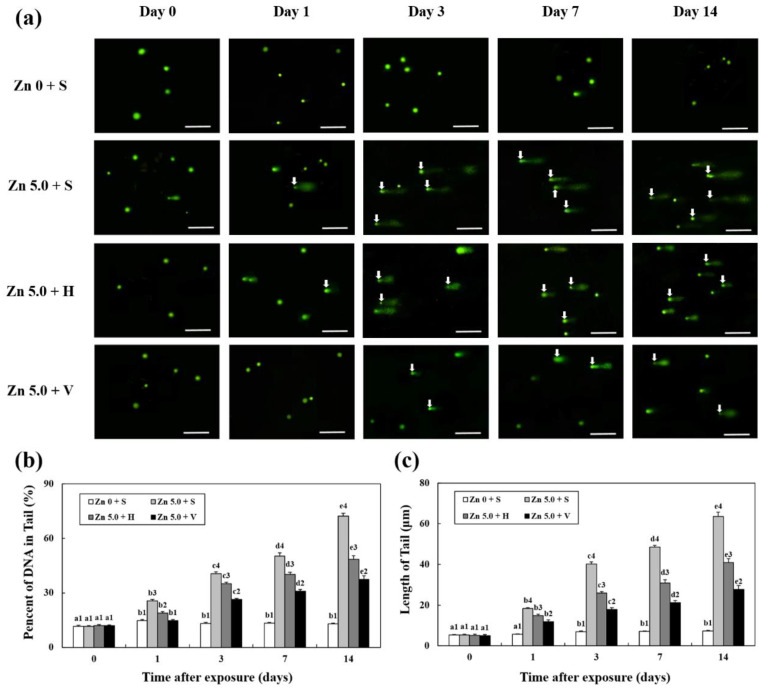
(**a**) Comet assay images and comet assay parameters, (**b**) tail length, and (**c**) DNA percentage in the tail for 14 days. Damaged nuclear DNA (DNA breaks) of the liver cells were stained with SYBR-gold. White arrows indicate damaged nuclear DNA (DNA breaks) in the cells (scale bars = 50 μm). Different letters indicate significant differences among goldfish exposed to the same Zn concentration and water hardness for different exposure time (*p* < 0.05). Different numbers indicate significant differences among goldfish exposed to different Zn concentrations and water hardness for the same duration (*p* < 0.05). Values indicate means ± SD (*n* = 4).

Following treatment with different Zn concentration and water hardness levels (soft, hard, and very hard), DNA damage in the liver tissue of goldfish was analyzed using 100 randomly selected cells for 14 days (Figure 5). Tail length and DNA content increased with the number of days of Zn exposure. When the water hardness was high, the length of the tail was short (i.e., day 7: Zn 5.0 + S; 48.6 ± 0.8; Zn 5.0 + H; 30.9 ± 1.5; Zn 5.0 + V; 21.2 ± 1.0, *p* < 0.05). Moreover, the DNA content in the tail was low with low water hardness in each group on the same exposure day (i.e., day 7: Zn 5.0 + S; 50.2 ± 1.8; Zn 5.0 + H; 40.1 ± 1.2, Zn 5.0 + V; 30.9 ± 1.0, *p* < 0.05). 

## 4. Discussion

When fish are exposed to high concentrations of Zn, it accumulates in the body and acts as a toxic substance that induces oxidative stress. However, according to a previous study, an increase in water hardness reduced the toxicity of high Zn concentrations [20]. In addition, it has been reported that water hardness decreased heavy metal accumulation in the tissues of tilapia *Oreochromis niloticus*, a freshwater fish exposed to a high water hardness environment, as well as the activity of antioxidant enzymes in response to heavy metal (Cd, Cu) exposure [24,25]. However, in these studies, only changes in the activity of metal accumulation and antioxidant enzymes were observed during fish survival. In this study, the changes in antioxidant enzyme activity as well as cell death and DNA damage caused by oxidative stress were analyzed to determine whether the stress of fish was reduced depending on the hardness of water exposed to high concentrations of Zn.

In this study, SOD and CAT activities were increased in goldfish *C. auratus* exposed to high concentrations of Zn, but their activities were decreased in the groups with high water hardness. Zikić et al. [26] reported that SOD activity increased when goldfish *C. auratus* were exposed to Cd, a heavy metal. Atli et al. [27] reported that CAT activity in the liver increased when tilapia *O. niloticus* was exposed to Zn. In addition, according to Saglam et al. [25], SOD and GPX activities were reduced in the CaCO_3_ addition group in a heavy-metal exposure environment. The results of this study are consistent with the activity patterns of antioxidant enzymes shown in our study. Based on this, it is concluded that water hardness works effectively to reduce oxidative stress in goldfish *C. auratus* caused by Zn, which is a heavy metal.

Using Mn-SOD-type, which is effective in removing O_2_^−^ generated as a by-product of oxidative phosphorylation, the change in SOD mRNA expression in liver tissue was observed through in situ hybridization. The SOD mRNA expression signal was expressed near the cell nucleus but not in the cytoplasm of hepatocytes. This result was similar to that of Orbea et al. [28], who reported that immunostaining of Mn-SOD was observed in hepatocytes and cholangiocytes around the portal vein of gray mullet *Mugil cephalus*. In addition, in this study, all 5.0 mg/L Zn groups showed high SOD activity. This means that the SOD level increased with high Zn concentration in hepatocytes. When goldfish were exposed to Zn, it was visually confirmed that an antioxidant reaction to remove ROS took place in the liver. Moreover, it was visually confirmed that SOD expression decreased as water hardness increased, even when exposed to the same concentration of Zn. According to these findings, it is determined that the decrease in the antioxidant reaction occurring in the liver of the goldfish was due to high water hardness, which reduced the ROS level generated due to the toxicity of high concentrations of Zn.

Although ROS are reduced due to antioxidant mechanisms, including the activity of antioxidant enzymes, ROS that are not sufficiently stabilized by these antioxidant actions eventually induce apoptosis through lipid peroxidation and caspase protein activation. In this study, plasma H_2_O_2_ and LPO levels increased with increasing Zn concentration and exposure time. This result was similar to that of Choi et al. [29], who demonstrated that H_2_O_2_ and LPO levels increased as a result of exposing the goldfish *C. auratus* to selenium (3 and 4 mg/L, respectively).

In this study, it was observed that H_2_O_2_ and LPO levels decreased as hardness increased, even in the experimental group with the same Zn concentration and exposure time. The results of this study showed that H_2_O_2_ and LPO levels increased with the passage of time after exposure to high concentrations of Zn, suggesting that significant accumulation of ROS occurred in the goldfish body. In addition, a decrease in H_2_O_2_ and LPO levels indicates that water hardness inhibits the generation of ROS.

Caspase-9 mRNA expression was analyzed to confirm the occurrence of apoptosis. When goldfish were exposed to Zn for 7 and 14 days, the expression level of Caspase-9 mRNA was increased. In the zebrafish *Danio rerio*, it was reported that the p53 gene, an apoptosis-related gene associated with the caspase-9 pathway, was activated and induced apoptosis due to ROS generated by exposure to Cu [30]. Jiao et al. [31] reported that exposing the common carp *Cyprinus carpio* to chlorpyrifos, a toxic insecticide component, activated caspase-9 due to ROS generated in the body, and induced apoptosis. Likewise, in the results of this study, the increase in caspase-9 following Zn exposure for 7 and 14 days can be viewed as a disproportion to the increase in ROS, and it is consistent with results of previous studies that reported that occurrence of ROS induces apoptosis. In addition, when comparing groups with the same Zn concentration and exposure period, the decrease in caspase-9 with increasing hardness is thought to be due to the decrease in ROS caused by water hardness.

A comet assay was performed in this study to confirm that DNA damage is a sign of apoptosis [32]. As a result, DNA damage increased with increasing Zn concentration and exposure period. This is similar to the findings of Kousar and Javed [33], who discovered that exposure of rohu *Labeo rohita*, mori *Cirrhina mrigala*, thaila *Catla catla*, and grass carp *Ctenopharyngodon idella* to various Zn concentrations (corresponding to 17%, 25%, 33%, and 50%, respectively, of the hemi-lethal Zn concentration that varied for each fish) was consistent with the finding that DNA damage increased with increasing Zn concentration. When comparing the same Zn concentration and exposure period groups, it was found that DNA damage decreased as water hardness increased. This was similar to the results of this study, in which the expression level of Caspase-9, an apoptosis-related gene, decreased as water hardness increased. These results confirmed that high concentrations of Zn accelerated apoptosis, whereas high water hardness alleviated the rate of apoptosis.

In this study, an increase in antioxidant enzyme activity was observed when goldfish were exposed to Zn concentrations of at least 1.0 mg/L. This is presumed to be due to the antioxidant enzymes acting as a defense mechanism for Zn to generate ROS in the body to stabilize ROS. However, it was observed that the concentrations of H_2_O_2_ and LPO continued to increase with Zn concentration and exposure time despite the active antioxidant response. This means that in the case of H_2_O_2_ and LPO, which were increased by exposure to Zn for a period of 14 days, antioxidant enzyme activity alone was insufficient to stabilize ROS and restore the antioxidant defense mechanism. In addition, while Zn exposure for 7 and 14 days increased the expression level of caspase-9, Zn exposure for 1 and 3 days did not appear to induce caspase-9 expression and thus did not generate enough ROS to cause apoptosis. Therefore, it seems that the antioxidant reaction that occurs for a relatively short period of 1 and 3 days can reduce the amount of ROS in the body to prevent apoptosis. However, it appears that if Zn exposure is prolonged, it will be difficult to reach the level of the antioxidant response sufficient to control the amount of ROS.

In our previous study, exposing goldfish to up to 3.0 mg/L of Zn, the stress-reducing effect of Zn through water hardness was confirmed [21]. Unlike previous studies, goldfish were exposed to up to 5.0 mg/L of Zn in this study. Then, we investigated whether the positive effect of water hardness, which reduces zinc toxicity in a short time, is applied even when exposed to Zn conditions exceeding the concentration that can cause lethality in freshwater fish [34]. As a result, it was confirmed that there were no deaths despite exposure to Zn concentrations that could cause lethality, and that a water hardness of at least 90 mg/L could reduce oxidative stress due to high Zn concentration.

## 5. Conclusions

In summary, (1) reducing the absorption rate of heavy metals (Zn) in goldfish and high water hardness (high CaCO_3_ concentration) appear to protect fish from toxicity caused by Zn. (2) It is believed that a water hardness of at least 270 mg/L CaCO_3_ is effective for effectively maintaining the antioxidant system of fish because the high level of oxidative stress caused by Zn was reduced as the concentration of water hardness increased.

The results obtained in this study are expected to be useful for investigating the relationship between the imbalance of the fish antioxidant system against heavy metals and environmental factors, such as water hardness and water quality.

## Figures and Tables

**Table 1:** Primers used for qPCR amplification and in situ hybridization.

Genes (Accession No.)	Primer	DNA Sequences
For qPCR		
Caspase-9 (XM026241892)	Forward	5′-CCA GGA CAT GAT CGA TGA AA-3′
	Reverse	5′-AGT TTC ACG CAG ACA CTC CA-3′
β-actin (LC382464)	Forward	5′-TTC CCT TGC TCC TTC CAC CA-3′
	Reverse	5′-TGG AGC CAC CAA TCC AGA CA-3′
For in situ hybridization		
Mn-SOD (KM065388)	Forward	5′-AGC ACC ATG CGA CTT ATG TC-3′
	Reverse	5′-CCC AGT TCA CAA CAT TCC AG-3′

**Table 2:** Water hardness (CaCO_3_ mg/L) during the experiment.

	Zn 0	Zn 1.0	Zn 2.0	Zn 5.0
Soft	90.1 ± 1.6	91.6 ± 1.8	91.4 ± 1.3	91.2 ± 1.3
Hard	271.1 ± 1.6	268.8 ± 4.0	269.6 ± 4.4	273.2 ± 3.4
Very hard	448.8 ± 4.2	449.2 ± 4.2	445.4 ± 5.4	447.2 ± 9.3

## Data Availability

All relevant data are within the manuscript.
